# Peach RNA viromes in six different peach cultivars

**DOI:** 10.1038/s41598-018-20256-w

**Published:** 2018-01-30

**Authors:** Yeonhwa Jo, Sen Lian, Hyosub Chu, Jin Kyong Cho, Su-Hyun Yoo, Hoseong Choi, Ju-Yeon Yoon, Seung-Kook Choi, Bong Choon Lee, Won Kyong Cho

**Affiliations:** 1 Research Institute of Agriculture and Life Sciences, College of Agriculture and Life Sciences, Seoul National University, Seoul, 08826 Republic of Korea; 2 College of Crop Protection and Agronomy, Qingdao Agricultural University, Qingdao, Shandong 266109 China; 3 The Taejin Genome Institute, Hoeongseong, Gadam-gil 61, 25239 Republic of Korea; 4 Virology Unit, Department of Horticultural Environment, National Institute of Horticultural and Herbal Science, RDA, Wan-Ju, 565-852 Republic of Korea; 5 Crop Foundation Division, National Institute of Crop Science, RDA, Wanju, 55365 South Korea

## Abstract

Many recent studies have demonstrated that several known and unknown viruses infect many horticultural plants. However, the elucidation of a viral population and the understanding of the genetic complexity of viral genomes in a single plant are rarely reported. Here, we conducted metatranscriptome analyses using six different peach trees representing six individual peach cultivars. We identified six viruses including five viruses in the family *Betaflexiviridae* and a novel virus belonging to the family *Tymoviridae* as well as two viroids. The number of identified viruses and viroids in each transcriptome ranged from one to six. We obtained 18 complete or nearly complete genomes for six viruses and two viroids using transcriptome data. Furthermore, we analyzed single nucleotide variations for individual viral genomes. In addition, we analyzed the amount of viral RNA and copy number for identified viruses and viroids. Some viruses or viroids were commonly present in different cultivars; however, the list of infected viruses and viroids in each cultivar was different. Taken together, our study reveals the viral population in a single peach tree and a comprehensive overview for the diversities of viral communities in different peach cultivars.

## Introduction

The peach is a kind of popular stone fruits in the world. Peach [*Prunus persica* (L.) Batch] belongs the genus *Prunus* which includes almond, apricot, cherry, and plum^1^. In Korea, the peach is the third fruit tree, which is widely cultivated after apple and mandarin. Diverse peach cultivars with different harvest times are being cultivated due to their short storage times. Peach cultivars are clonally propagated to maintain fruit traits via grafting. Viruses and viroids infecting peach trees can be transmitted by grafting using virus-infected materials. To date, more than 20 different viruses infecting peach trees have been identified. Of known viruses infecting peach trees, *Apple chlorotic leaf spot virus* (ACLSV; genus *Trichovirus*), *Plum pox virus* (PPV; genus *Potyvirus*), *Prunus necrotic ringspot virus* (PNRSV; genus *Ilarvirus)*, and *Plum bark necrosis stem pitting-associated virus* (PBNSPaV; genus *Ampelovirus*)^2–5^. Furthermore, the peach is susceptible to two different viroids including *Hop stunt viroid* (HSVd; genus *Hostuviroid*) and *Peach latent mosaic viroid* (PLMVd; genus *Pelamoviroid*)^6,7^. Although most peach trees infected by HSVd and PLMVd are asymptomatic, some PLMVd isolates cause severe disease symptoms such as albinism, leaf mosaics and blotches^8^.

Thus far, the identification of the viruses and viroids that infect plants relies on collecting samples displaying viral disease symptoms followed by direct detection using RT-PCR and ELISA^9,10^. However, such approaches have only led to the identification of known viruses and viroids, rather than novel viral pathogens. Several next-generation sequencing (NGS)-based approaches have been conducted to overcome traditional approaches, which has resulted in the identification of known and novel viruses^11–13^. For example, two recent studies using NGS identified novel viruses in nectarines that might be responsible for stem-pitting disease symptoms^14,15^. Moreover, a recent study using NGS reported a novel virus referred as Peach leaf pitting-associated virus (PLPaV) in the genus *Fabavirus*, which is responsible for a leaf symptom^16^. Furthermore, peach-assoicated luteovirus and Peach virus D (PeVD) are also newly identified using NGS^17,18^.

The term “virome” is defined as the genomes of all the viruses inhabiting a specific organism or environment^19^. In fact, many horticultural plants that are usually clonally propagated are reservoirs of a large variety of viruses and viroids. However, although studies associated with a single virus infection in a specific plant are numerous, viromes or viral populations in a particular plant have not been intensively examined. Thus far, only a limited number of studies have revealed viromes in specific plants such as grapevines^20–22^, sweet potato^23^, and pepper^24^. Furthermore, NGS-based approaches enable the assembly of viral genomes and reveal single nucleotide variations of present viruses^24–26^.

Although peach is an important fruit tree; however, the studies associated with viruses and viroids infecting peach trees in Kore are limited. In this study, we conducted a peach RNA virome study in six different peach cultivars, which are commercially important in Korea. We identified both known viruses and viroids and a novel virus. In addition, the peach RNA virome reveals host specific viral communities in different peach cultivars, but unique viral communities of each peach plant associated with variety of factors.

## Results

### Preparation of samples, library construction, and RNA-Seq for metatranscriptome analyses

**Figure 1:**
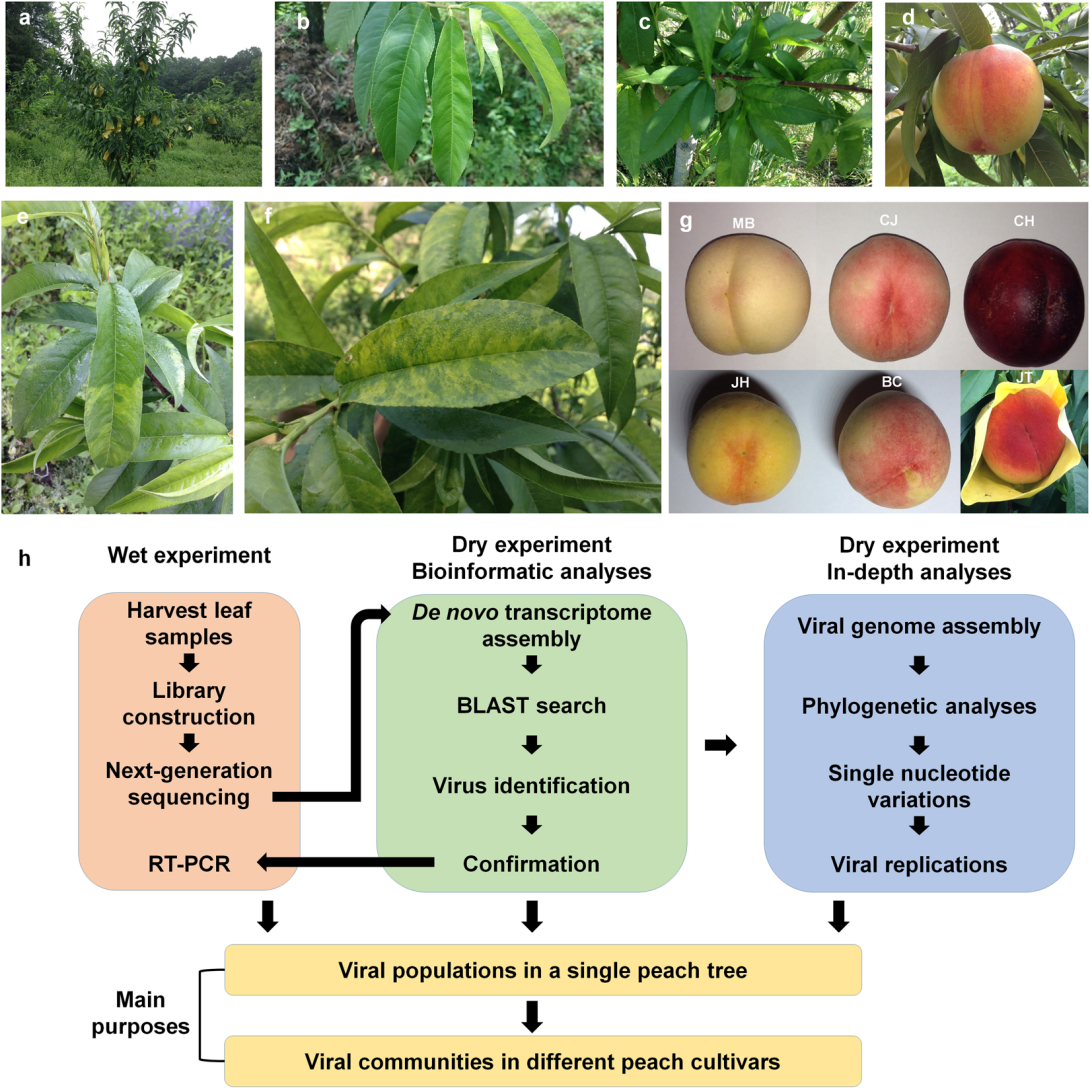
The diverse phenotypes of the different peach cultivars and the experimental scheme. (**a**) A mature peach tree of the JT cultivar showing vigorous growth, (**b**) Healthy peach leaves without viral disease symptoms, (**c**) A peach branch with a young peach fruit and peach leaves showing viral disease symptoms, (**d**) A mature peach fruit without viral disease symptoms, (**e** and **f**) Peach leaves with viral disease symptoms. Peach fruits obtained from six examined peach trees. In general, the peach fruits were wrapped in a yellow envelope for the production of high quality fruit, (**h**) Experimental scheme of a peach virome study composed of wet experiment and dry experiment.

We initially examined an infection of *Apple chlorotic leaf spot virus* (ACLSV; genus *Trichovirus*) and two viroids (HSVd and PLMVd) in various peach trees by RT-PCR to study the peach virome in different peach cultivars. The RT-PCR results demonstrated that most peach trees were infected by at minimum either ACLSV or PLMVd. Most peach trees used in this study grew vigorously (Fig. 1a) and did not display any severe disease symptoms (Fig. 1a,d); however, some young developing leaves in specific cultivars showed very mild disease symptoms such as chlorotic local lesions, leaf spot, pale green coloring, and yellowing (Fig. 1e,f). Regardless of viral disease symptoms, all six trees produced high quality peach fruits (Fig. 1g). Out of the peach trees infected by at least one virus or viroid, we selected six different peach trees to represent six different peach cultivars, which are economically important in Korea. As shown in the experimental scheme (Fig. 1h), we harvested leaves from a single tree and subjected them to the construction of poly-A-enriched libraries. Six different libraries representing six different peach trees were pair-end sequenced by HiSeq. 2000 system.

## Identification of viruses and viroids from peach transcriptomes

The raw data sequenced by RNA-Seq from each library was deposited in an SRA database with respective accession number (Table S1). First, six peach transcriptomes were individually *de novo* assembled using the Trinity program^27^. The number of obtained transcripts ranged from 110,477 (JT) to 146,623 (BC) (Table S2). The values for contig N50 ranged from 1,594 bp to 1,983 bp.

The assembled contigs from each library were blasted against a house-made viral genome database to determine virus-associated contigs. Ninety-two virus-associated contigs were identified after removing endogenous virus-associated sequences (Table S3). Although initial MEGABLAST results identified nine viruses including two viroids in total (Table S4), several intensive bioinformatics analyses such as viral genome assembly and sequence alignment demonstrated that contigs associated with *Apple green crinkle associated virus* (AGCAV; genus *Foveavirus*) were partial sequences for Asian prunus virus (APV; genus *Foveavirus*), which can be further divided into APV1 and APV2. Moreover, contigs associated with *Maize rayado fino virus* (MRFV) and *Anagyris vein yellowing virus* (AVYV), belonging to the family *Tymoviridae* were identified as partial sequences or nearly complete genome sequences for a novel virus.

**Figure 2:**
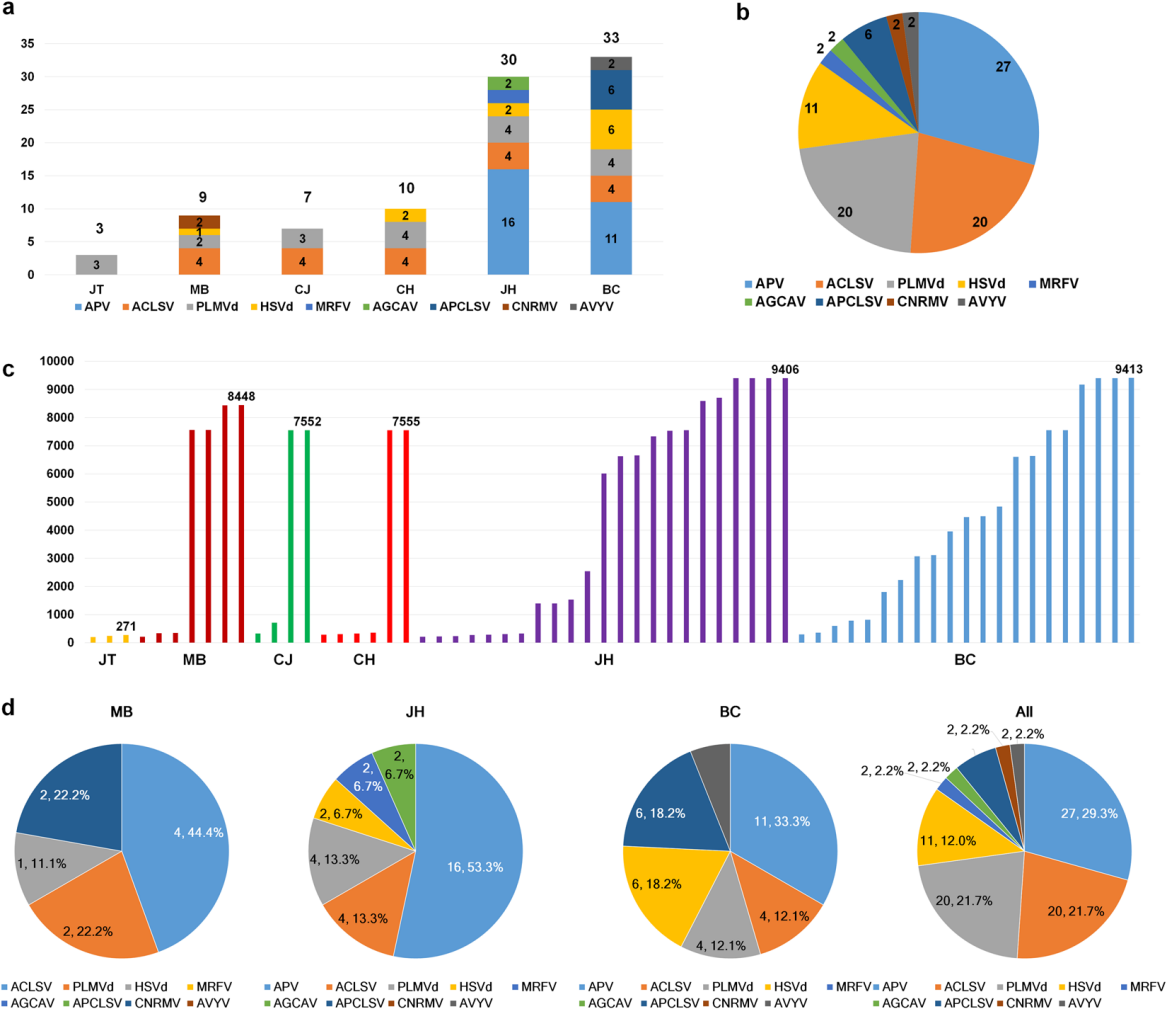
The identification of the viruses and viroids infecting the peach trees using transcriptome analyses. (**a**) The number of viral contigs associated with identified viruses and viroids in each peach transcriptome. We conducted the initial identification of viruses and viroids via MEGABLAST search against the plant virus database using the assembled contigs from each peach transcriptome. The number on each bar indicates the total number of virus-associated contigs in each peach transcriptome. (**b**) The portion of identified viruses and viroids based on the number of contigs associated with each virus or viroid. (**c**) The size of contigs associated with each identified virus or viroid. The number indicates the size of the longest viral contig for each peach transcriptome. (**d**) The portion of identified viruses and viroids in three different peach cultivars: MB, JH, and BC, and all six cultivars based on number of contigs associated with each virus or viroid.

JT cultivar was only infected by PLMVd, while JH and BC cultivars were infected by four viruses and two viroids (Fig. 2a). The most frequently identified virus in the six peach cultivars was ACLSV, which resided in five cultivars except JT (Table S4). In addition, PLMVd was present in all six cultivars (Table S4). The number of identified viruses and viroids in each peach transcriptome correlated with the number of virus- and viroid-associated contigs, respectively. JT only contained three contigs associated with PLMVd, while JH and BC respectively possess 30 and 33 virus-associated contigs (Fig. 2a). Of the 92 contigs associated with viruses and viroids, the APV (APV1 and APV2)-associated contigs (27 contigs) were dominant, followed by ACLSV (20 contigs) and PLMVd (20 contigs)-associated contigs (Fig. 2b). The length of virus-associated contigs ranged from 206 bp to 9,413 bp (Fig. 2c). There were 28 contigs, in which sequence length was more than 6,000 bp, indicating that they might be *de novo* assembled partial or nearly complete genomes for identified RNA viruses and viroids. We examined the portion of virus-associated contigs in individual cultivars (Fig. 2d). For example, ACLSV (four contigs) were dominant, followed by PLMVd (two contigs) and *Cherry necrotic rusty mottle virus* (CNRMV; genus *Robigovirus*) (two contigs) in MB cultivar. APV-associated contigs (16 contigs) derived from APV1 and APV2 were dominant in JH and similarly, 11 contigs derived from APV2 were enriched in BC. By combining all virus-contigs, APV (27 contigs), ACLSV (20 contigs), PLMVd (20 contigs), and HSVd (11 contigs) were highly present. As a result, we identified six viruses: APV1, APV2, ACLSV, APCLSV, CNRMV, and one novel virus, and two viroids: HSVd and PLMVd, from six peach transcriptomes.

## The de novo assembly of viral genomes using transcriptome data

**Figure 3:**
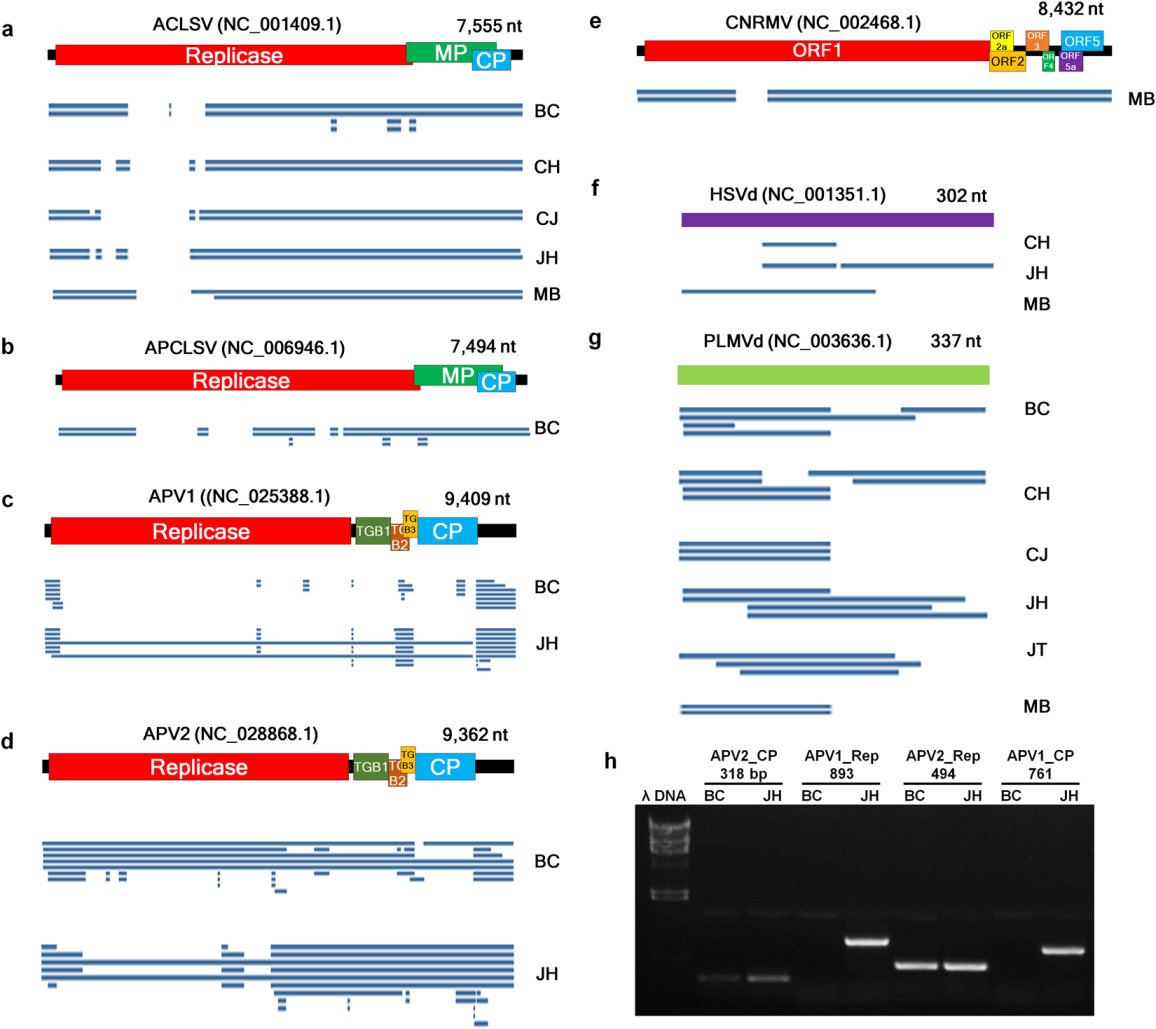
Genome assembly of identified viruses and viroids using peach transcriptome data. We obtained the complete or nearly complete genome of several viruses and viroids. The genome organization of assembled viruses with accession number and the size of corresponding reference viral genomes and the alignment of associated contigs derived from individual peach transcriptome on each virus reference genome for ACLSV (**a**), APCLSV (**b**), APV1 (**c**), APV2 (**d**), CNRMV (**e**), HSVd (**f**), and PLMVd (**g**). Abbreviations for open reading frames: movement protein (MP), coat protein (CP), triple gene block (TGB). In the case of viroids with a circular RNA genome, we used a linear RNA genome for the alignment of contigs associated with each viroid. (**h**) RT-PCR results showing amplification of partial sequences for APV1 and APV2 using APV1- and APV2-specific primers in BC and JH peach cultivars.

We were able to assemble the complete or nearly complete genomes of several viruses and viroids using transcriptome data. For this, we mapped virus-associated contigs on each reference viral genome (Fig. 3a–g). Some contigs associated with APV1 and APV2 covered nearly the complete genomes of two viruses (Fig. 3c,d). However, some sequence regions in the genomes of identified viruses and viroids were partially mapped by the virus associated contigs (Fig. 3a,b,e,f, and g). To obtain additional viral contigs, we additionally conducted *de novo* transcriptome assembly using the Velvet program^28^. Using contigs obtained by the Trinity and Velvet programs, we could assemble the nearly complete genomes for several viruses and viroids (Table S5). Five viral genomes were obtained for ACLSV (Fig. 3a and Table S5). In addition, we obtained a nearly complete genome of APCLSV from the BC cultivar (Fig. 3b) as well as two genomes for APV1 (Fig. 3c) and APV2 from BC and JH cultivars (Fig. 3d), respectively. A nearly complete genome of CNRMV was obtained from the MB cultivar (Fig. 3e). In the case of HSVd, we only obtained partial sequences from the CH, JH, and MB cultivars (Fig. 3f). Moreover, three complete RNA genomes for PLMVd were obtained from BC, CH, and JH (Fig. 3g). Due to high sequence similarity of APV1 and APV2, we conducted RT-PCR using APV1- and APV2-specific primers. Both the sequence data and RT-PCR results confirmed that APV1 and APV2 were infected in both BC and JH while only APV1 was infected in JH (Fig. 3h).

## Identification of a novel virus from two peach transcriptomes

**Figure 4:**
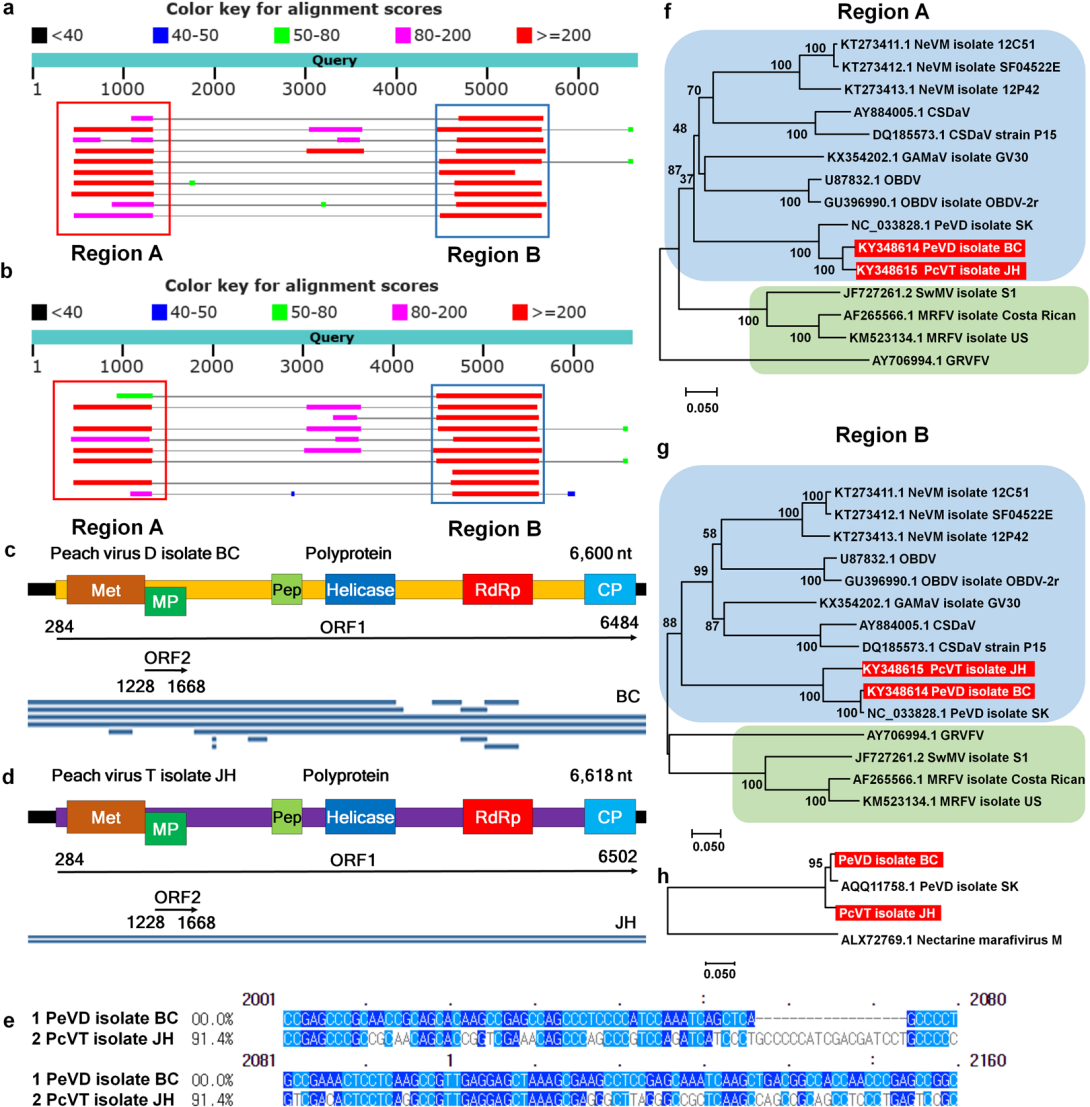
Identification of a novel peach virus from two peach transcriptomes. (**a**) Visualization of BLAST result with the obtained PeVD isolate BC genome sequence. Red and blue boxes indicate region A and region B sequences, respectively, which show strong sequence similarity to other known viruses. (**b**) Visualization of BLAST results with the obtained PcVT isolate JH genome sequence. (**c**) Genome organization and the alignment of contigs associated with PeVD on the PeVD genome for the PeVD isolate BC. Two open reading frames (ORF)s: ORF1 and ORF2 are indicated by arrow lines with corresponding sequence positions. (**d**) Genome organization and the alignment of contigs associated with PcVT on the PcVT genome for PcVT isolate BC. (**e**) Comparison of nucleotide sequences between PeVD isolate BC and PcVT isolate JH. Phylogenetic trees of two PeVD isolates and a PcVT isolate and the associated known viruses based on regions A (**f**) and B (**g**) sequences. (**h**) Phylogenetic tree of two PeVD isolates and a PcVT isolate using polyprotein sequences. *Nectarine marafivirus M* was used as an outgroup.

Most virus-associated contigs in this study were matched to sequences of known viruses and viroids. Some contigs in JH and BC cultivars showed high sequence similarity to known viruses, including MRFV, AVYV, *Nectarine marafivirus M*, *Grapevine Syrah Virus-1*, and *Grapevine asteroid mosaic-associated virus* (Fig. 4a,b, Table S6); however, their sequence coverages were very low and only two sequence regions, A and B, displayed strong sequence similarity to known viruses. This result suggests that those contigs might be sequences derived from a novel virus. The lengths of two contigs from JH and BC cultivars were more than 6,000 bp. Based on the *de novo* assembled contigs and RT-PCR, we obtained two nearly complete viral genomes. These two viral genomes were blasted against the nucleotide database (NT) in NCBI, and the BLASTN results showed that a viral genome from a BC cultivar shared a 95% nucleotide identity with the recently identified PeVD^18^, while a viral genome from the JH cultivar shared a 88% nucleotide identity with the PeVD. Since the viral genome from BC shared a strong sequence similarity to the PeVD, we have named it “PeVD isolate BC.” However, the novel viral genome from the JH cultivar was different from PeVD. Thus, we have tentatively named it “Peach virus T (PcVT) isolate JH” (Fig. 4c,d). The genomes of PeVD isolate BC and PcVT isolate JH demonstrated strong nucleotide sequence differences, such as 18 nucleotide deletions at positions 2057–2075 of the PeVD isolate BC compared to PcVT isolate JH (Fig. 4e). Both PeVD and PcVT encode two open reading frames (ORFs) (Fig. 4c,d); however, the presence of ORF2 in PeVD has not been reported in the previous study^18^. The ORF1 encodes a polyprotein that contains six conserved domains such as methyltransferase (Met), peptidase (Pep), helicase, RNA-dependent RNA polymerase (RdRp), and coat protein (CP), while ORF2 encodes movement protein (MP). We constructed two different phylogenetic trees using sequences from regions A and B to reveal the possible taxonomical classification of PeVD and PcVT (Fig. 4f,g). PeVD isolate BC and PcVT isolate JH as well as PeVD isolate SK were grouped together with other viruses such as *Citrus sudden death–associated virus* (CSDaV), *Grapevine asteroid mosaic associated virus* (GAMaV), *Nectarine virus M* (NeVM), and *Oat blue dwarf virus* (OBDV) belonging to the genus *Marafivirus* in the family *Tymoviridae*. Furthermore, a phylogenetic tree using the polyprotein of ORF1 clearly showed that PcVT isolate JH is different from PeVD (Fig. 4h). Thus, PcVT is a novel virus composed of a single-strand RNA genome belonging to the genus *Marafivirus* in the family *Tymoviridae*.

## The phylogenetic relationships of the identified viruses and viroids with other known viruses and viroids

**Figure 5:**
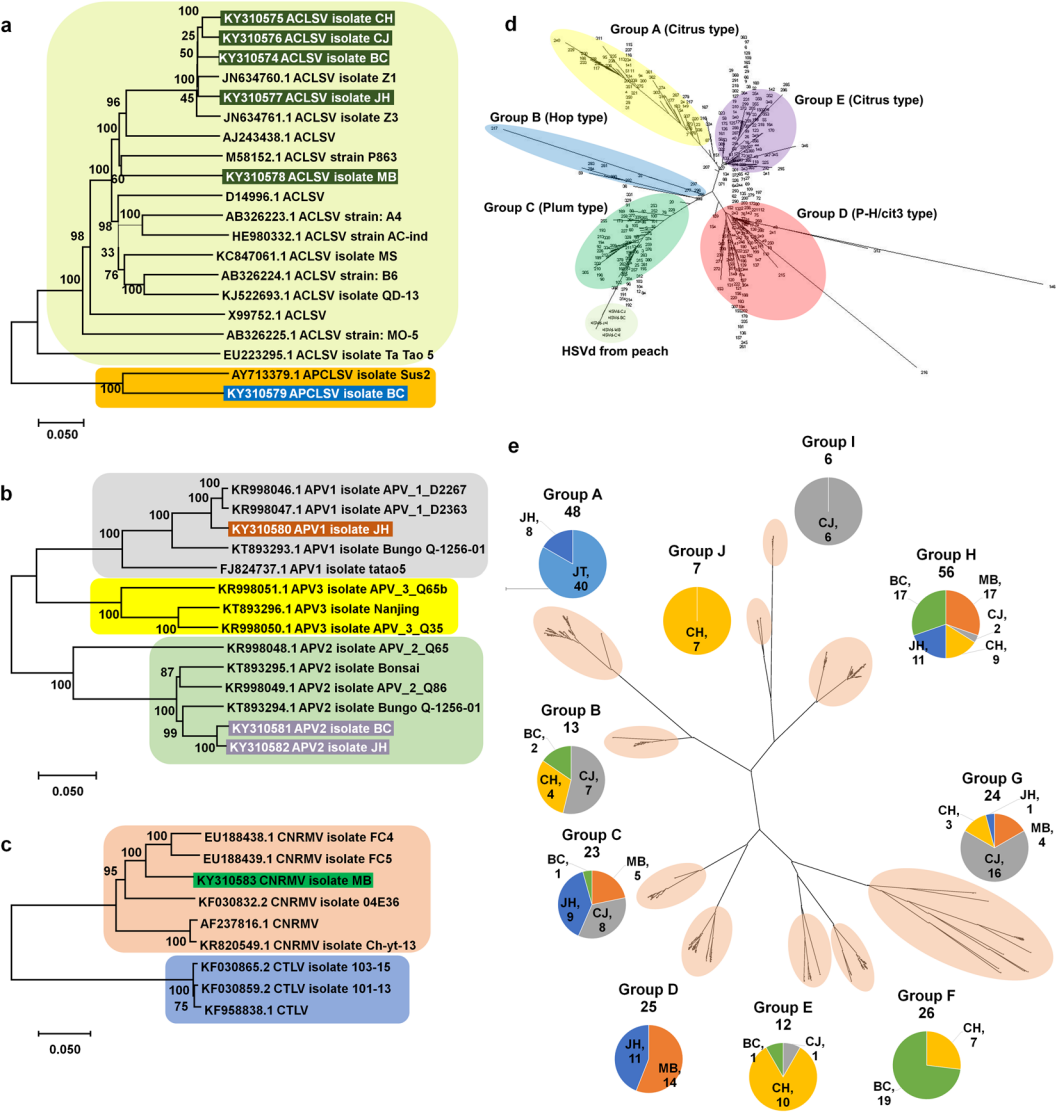
Phylogenetic analyses of identified viruses and viroids. (**a**) Phylogenetic relationships for five ACLSV isolates: CH, CJ, BC, JH, and MB, as well as APCLSV isolate BC in this study with known ACLSV and APCLSV genomes. (**b**) Phylogenetic relationships of APV1 isolate JH, APV2 isolate BC, and APV2 isolate JH with known APV1, APV2, and APV3 genomes. (**c**) The phylogenetic relationships of CNRMV isolate MB and known CNRMV and CTLV genomes. (**d**) The phylogenetic relationships of five HSVd isolates in this study with all HSVd genomes. The accession numbers for five HSVd genomes are KX371905–KX371909. (**e**) The phylogenetic relationships of 240 PLMVd genomes obtained via RT-PCR following by Sanger Sequencing in this study. The accession numbers of 240 PLMVd genomes are PLMVd-BC (KY355162–KY355201), PLMVd-CH (KY355202–KY355241), PLMVd-CJ (KT033033–KT033052 and KY355242–KY355261), PLMVd-JH (KY355262–KY355301), PLMVd-JT (KY355302–KY355341), and PLMVd-MB (KY355342–KY355381). The pie chart indicates the composition of identified PLMVd genome from each peach cultivar with the respective number of PLMVd genomes in each identified group.

We constructed phylogenetic trees to reveal the phylogenetic relationships of identified viruses and viroids with known other isolates. We subjected the assembled viral genome sequences in this study to a BLASTN search against the NT database in NCBI. We only used complete or nearly complete genome sequences of known viruses matched to the each assembled viral genome in this study for phylogenetic analyses. The BLAST results showed strong sequence similarity for ACLSV and *Apricot pseudo-chlorotic leaf spot virus* (APCLSV; genus *Trichovirus*) (Table S7). Therefore, we included both ACLSV and APCLSV in the construction of a phylogenetic tree, which was composed of five ACLSV isolates and one APCLSV isolate obtained from this study in addition to 13 known ACLSV isolates and one APCLSV isolate from GenBank in NCBI (Fig. 5a). A phylogenetic tree displayed two distinguishable groups: one with only ACLSV and one with APCLSV. Out of the five ACLSV isolates in our study, four ACLSV isolates were grouped together with ACLSV isolate Z1 and Z3 derived from peaches in China^3^. The ACLSV isolate MB was closely related to known ACLSV strain P863^29^. In the same way, APV1 and APV2 showed high sequence similarity based on BLASTN results (Table S8). Thus, we generated a phylogenetic tree composed of known APV1, APV2, and APV3 genomes, along with three genomes for APV1 and APV2 from this study (Fig. 5b). As expected, the phylogenetic tree exhibited three groups containing APV1, APV2, and APV3 isolates, respectively. The APV1 isolate JH was closely related to isolates APV_1_D2363 and APV_1_D2367. The genome sequences of APV2 isolate BC and JH were highly homologous and closely related to the APV2 isolate Bungo from Japan^30^. The phylogenetic tree composed of six CNRMV isolates and three CTLV isolates showed that CNRMV isolate MB (Table S9) was closely related to CNRMV isolate FC4 and FC5 from *Prunus serrulata* cultivar Gioko in Japan^31^.

Although we assembled two genomes for HSVd using transcriptome data, we conducted RT-PCR to obtain five complete genomes from five peach cultivars. Cloning followed by Sanger-sequencing produced five HSVd complete genomes (KX371905-KX371909). In general, the HSVd genome sequences were highly conserved. The five HSVd genomes and 382 HSVd variants from a recent study^32^ were subjected to phylogenetic tree construction. The phylogenetic tree using 387 HSVd genomes displayed five HSVd isolates in this study were grouped with Group C containing HSVd variants from plum; however, the five HSVd isolates from peach were grouped in a different clade (Fig. 5d).

We amplified 40 PLMVd genomes from each cultivar by RT-PCR using two different primer-pairs to reveal genetic diversity of PLMVd in different peach cultivars. In total, 240 PLMVd genomes were subjected to phylogenetic tree construction. The phylogenetic tree identified at least ten different groups of PLMVd genomes (Fig. 5e). The number of genomes in each group ranged from seven to 56 genomes. The group H (56 genomes) was the largest group followed group A (48 genomes), group F (26 genomes), and group D (25 genomes). Many groups contained genomes from different peach cultivars. For instance, Group A contained 48 genomes derived from JH and JT, while group H contained 56 genomes derived from five cultivars.

## Analyses of SNPs for individual viruses and viroids

**Figure 6:**
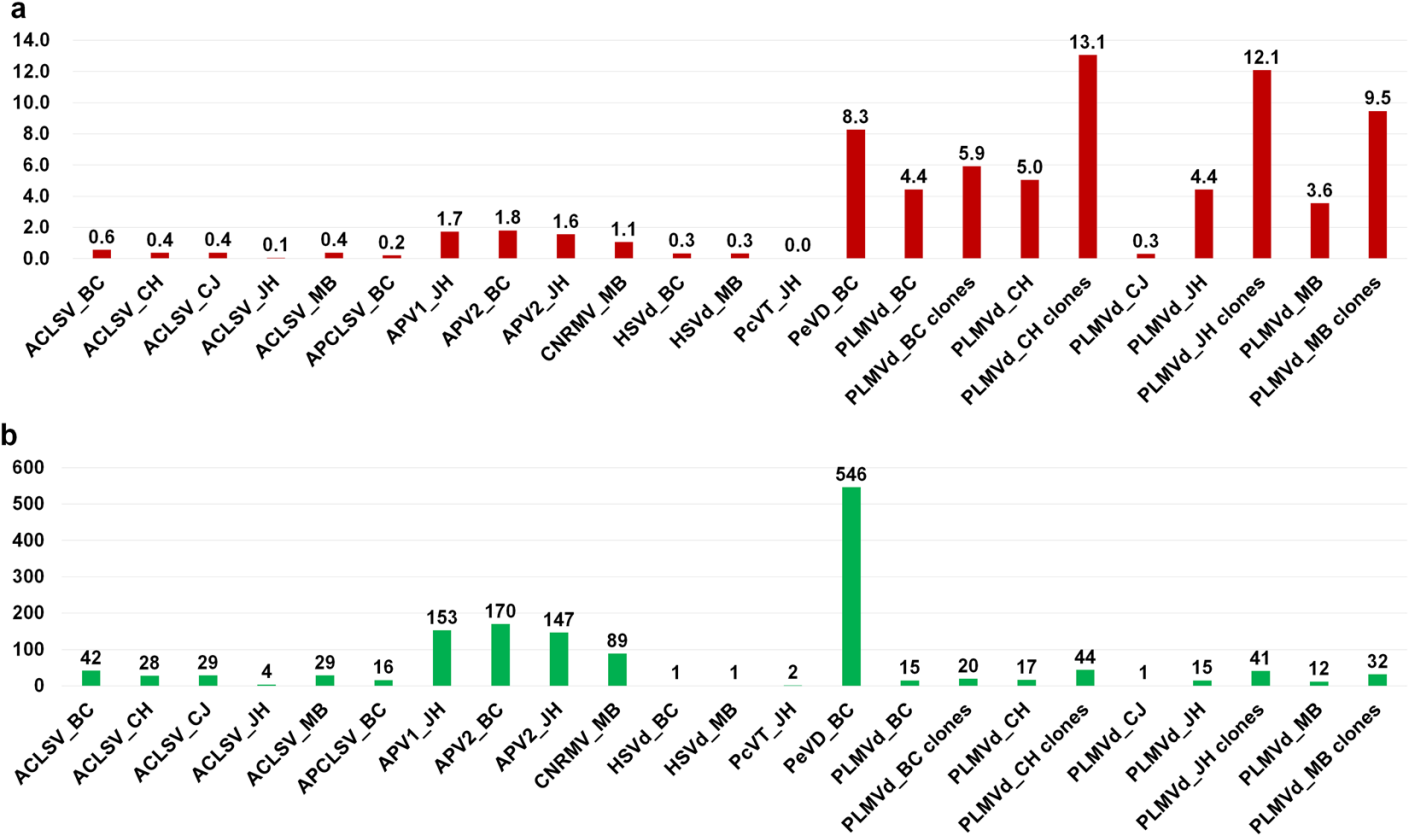
A summary of the identified SNPs for each virus or viroid in individual peach cultivars. The number of identified SNPs for each virus or viroid (**a**) and the percentage of SNPs for identified viruses in individual peach cultivars (**b**). The names of viruses and viroids are abbreviated; clones indicate that SNPs were obtained via RT-PCR followed by Sanger Sequencing.

In general, viruses and viroids with RNA genomes replicate with high mutation rates, which results in significant genetic diversity. In this study, we analyzed SNPs for the viruses and viroids in which we could assemble genomes from transcriptome data. We aligned raw sequence reads from each transcriptome on the each assembled viral genome and subjected to SNP identification. All detailed information associated with identified SNPs was described (Table S10). In the BC cultivar, SNPs for ACLSV, APCLSV, APV2, PeVD, and PLMVd were analyzed (Fig. S1) while SNPs for ACLSV, APV1, APV2, PcVT, and PLMVd were analyzed in the JH cultivar (Fig. S2). Many reads were aligned on the genomes of the corresponding virus genomes (Fig. S1a–d) while a relatively small number of reads were aligned on the PLMVd (Fig. S1e). In the case of PLMVd, we analyzed SNPs using both transcriptome data (Fig. S1e) and 40 PLMVd genomes amplified by RT-PCR followed by Sanger-sequencing (Fig. S1f). In a similar manner, SNPs for viruses and viroids in MB (Fig. S3), CJ, and CH (Fig. S4) were analyzed. We examined the number of identified SNPs in each viral genome (Fig. 6a). HSVd isolate BC, MB, and PLMVd CJ showed only one SNP, while PeVD isolate BC (546 SNPs) showed the largest number of SNPs. In addition, APV1 isolate JH (153 SNPs), APV2 isolate BC (170 SNPs), APV2 isolate JH (147 SNPs), and CNRMV isolate MB (89 SNPs) have relatively large numbers of SNPs. We next examined the mutation rate of identified viral genome in each transcriptome (Fig. 6b). PLMVd showed a high level of mutation rates ranging from 13.1% (for PLMVd isolate CH with 40 clones) to 3.6% (for PLMVd isolate MB). Among the examined viruses, although the PeVD isolate BC showed a high level of mutation rate (8.3%), the genome of PcVT isolate JH was not significantly mutated. In addition, APV1, APV2, and CNRMV showed relatively high levels of mutation rates compared to other viruses.

## Virus accumulation and copy number in a single peach tree

**Figure 7:**
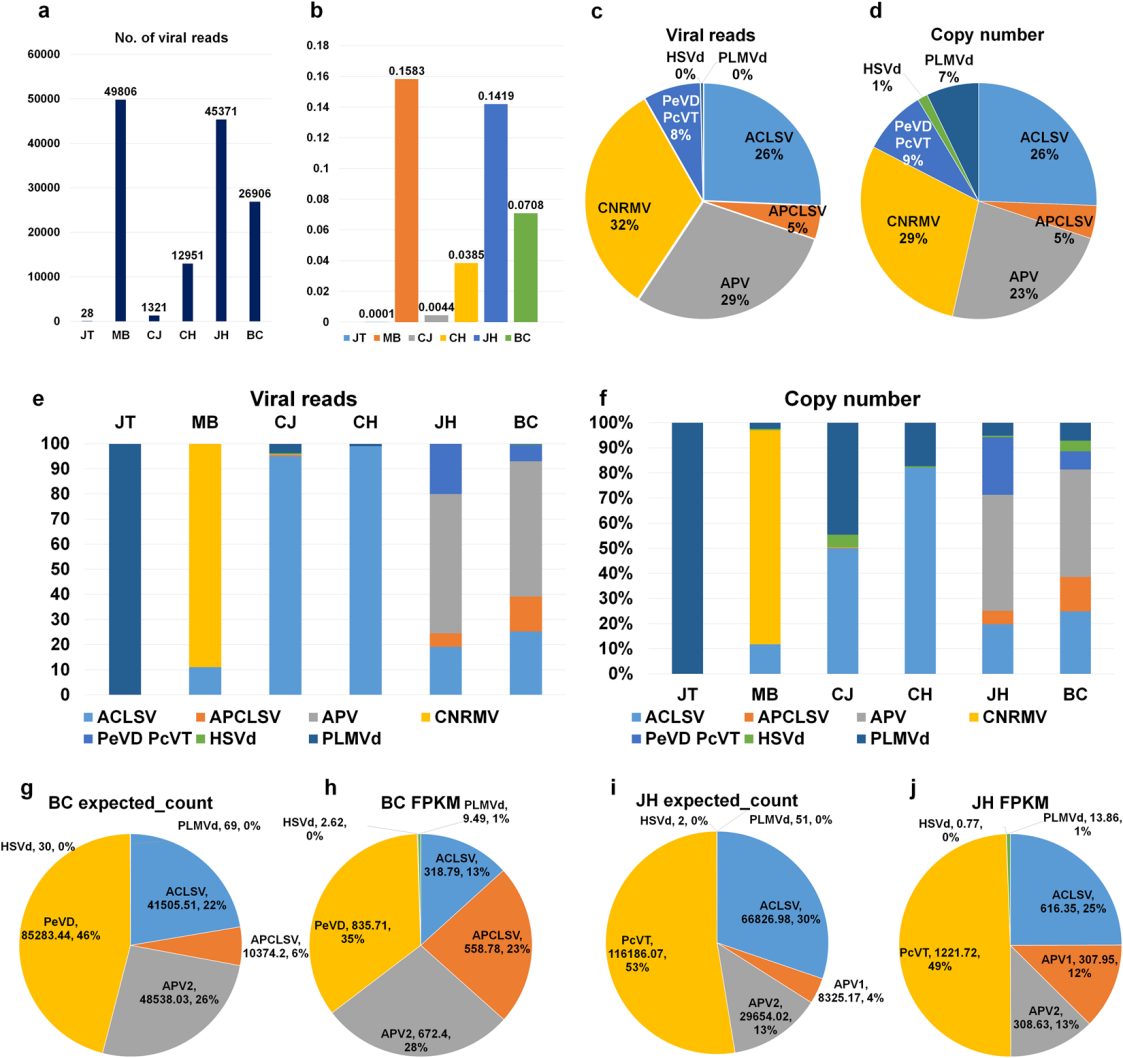
The virus accumulation and copy number for each identified virus or viroid in each peach transcriptome. (**a**) The number of sequenced reads associated with the identified viruses and viroids in each peach transcriptome. We blasted all read sequences in each cultivar with E-value 1e^−5^ as a cutoff against the reference genome sequences of identified viruses and viroids and calculated the number of matched reads. (**b**) The portion of virus-associated reads in each transcriptome was calculated as the number of viral reads divided by all sequenced reads. (**c**) The portion of identified viruses and viroids based on the number of virus-associated reads in all six peach transcriptomes. In the case of APV1 and APV2 as well as PeVD and PcVT, their sequenced reads were combined due to their high sequence similarity. (**d**) The portion of the identified viruses and viroids based on their copy numbers. The copy number was calculated as the number of identified sequence reads multiplied by 101 bp, then divided by the size of the identified virus genomes. (**e**) The portion of the identified viruses and viroids in each transcriptome based on the viral reads. (**f**) The portion of identified viruses and viroids in each transcriptome based on their copy number. The portion of identified viruses and viroids in the BC cultivar was based on (**g**) the calculated expected read count and (**h**) FPKM values. The portion of identified viruses and viroids in the JH cultivar based on (**i**) the calculated expected read count and (**j**) FPKM values. The expected read count and FPKM values were calculated using the Trinity program, as described in the methods section.

Although we designed our study for virus identification using RNA-Seq, most sequenced reads were derived from peach plants. The number of sequenced reads associated with identified viruses and viroids in each transcriptome varied. JT (28 reads) and CJ (1,321 reads) transcriptomes contained relatively low numbers of virus-associated reads, whereas MB (49,806 reads) and JH (45,371 reads) possessed a high number of virus-associated reads (Fig. 7a). Next, we analyzed the amount of virus accumulation in each transcriptome based on its read number, which was done by dividing the number of total reads by the number of virus-associated reads and multiplying them by 100. The amount of virus accumulation in each transcriptome was very low, ranging from 0.0001% (JT) to 0.1583% (MB) (Fig. 7b). Using only virus-associated reads, we calculated the accumulation of the individual virus in each transcriptome by dividing the number of total virus-associated reads by the number of individual virus-associated reads and multiplying them by 100 (Fig. 7c). CNRMV (32%) was the dominant virus, followed by two variants of Asian prunus viruses (29%), ACLSV (26%), both PeVD and PcVT (8%), and APCLSV (5%). The accumulation of the two viroids was extremely low. In addition, we calculated the copy number, also known as the sequence coverage, of each identified virus based on the number of virus-associated reads (Fig. 7d). The copy number was calculated by multiplying the number of individual virus-associated reads by 101 bp and dividing the result by the size of the virus genome. In our study, we assumed that the length of a sequenced read averaged 101 bp for paired-end sequencing using HiSeq. 2000. The results of the copy number were similar to those of virus accumulation except for HSVd (1%) and PLMVd (7%), in which the copy numbers were dramatically increased due to their small genome sizes. We examined the virus accumulation and copy number of each transcriptome (Fig. 7e and f). The dominant virus in each transcriptome was easily identified based on virus accumulation, such as PLMVd in JT, CNRMV in MB, ACLSV in both CJ and CH, and APV in JH and BC. However, based on the copy number, the portion of PLMVd was increased in CJ and CH. In order to determine the amount of viral RNA for each virus using transcriptome data, we used the FPKM method, which is frequently used for the expression profiling of host genes using RNA-Seq.^33^. We subjected two transcriptomes, BC and JH, to FPKM analysis. Based on the number of the expected read count (expected count), PeVD (46%) was the dominant in BC (Fig. 7g) while APV2 (28%) was dominant based on FPKM values (Fig. 7h). In JH, PcVT was again the dominant virus based on the expected count and FPKM values (Fig. 7i and j). However, the portion of APV1 was decreased based on the expected count compared to the FPKM values.

## Confirmation of presence of identified viruses and viroids by RT-PCR

**Figure 8:**
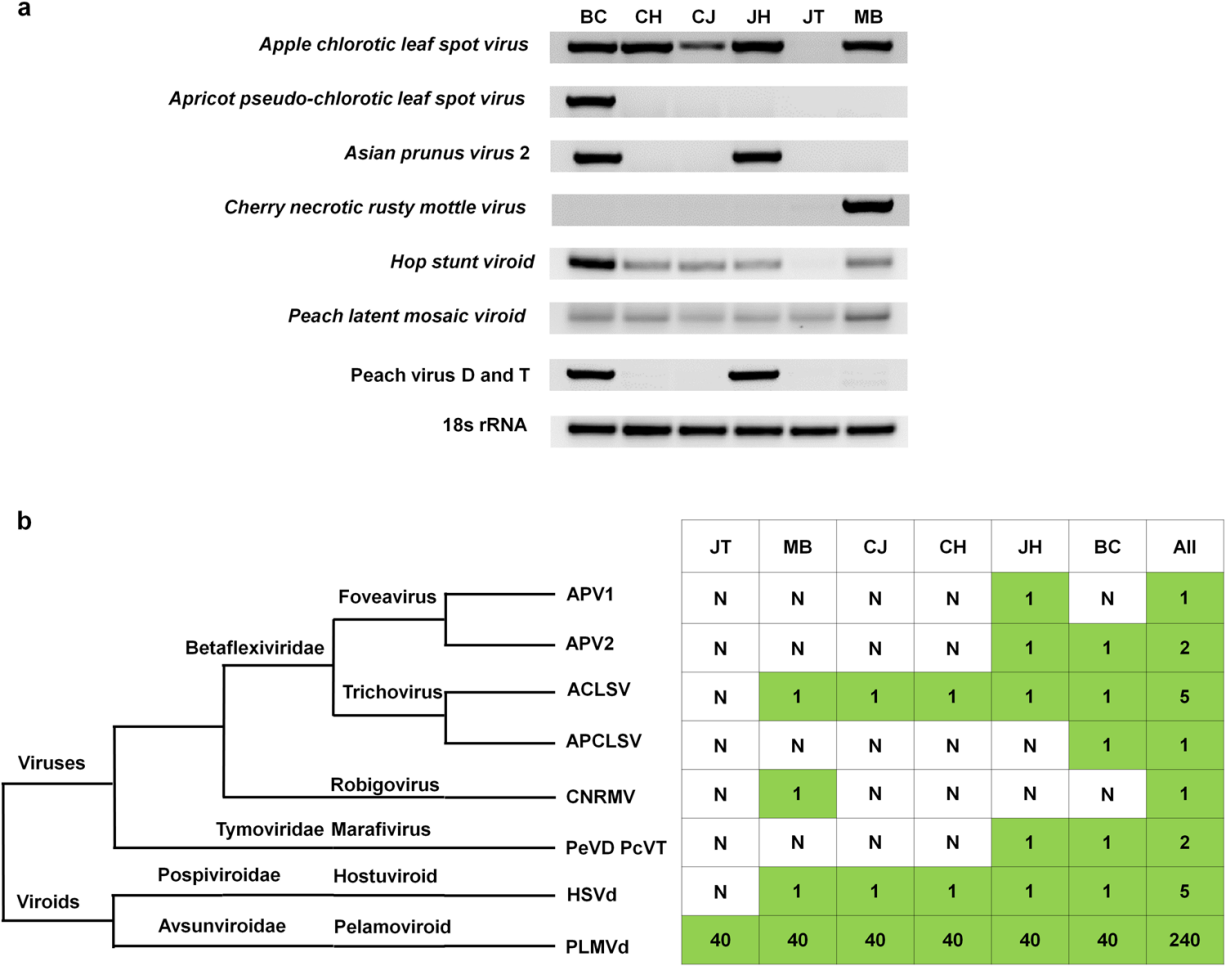
Confirmation of the identified viruses and viroids by RT-PCR and a summary of the identified viruses and viroids based on their taxonomy in each peach cultivar. (**a**) RT-PCR results with virus- and viroid-specific primers. The 18 s rRNA gene was used as a positive control. Full-length gels can be found in Supplementary Figure S5. (**b**) The taxonomy of identified viruses and viroids; N indicates no identification. The number indicates the number of viral genomes obtained from each cultivar in this study.

We conducted RT-PCR using each virus- and viroid-specific primer to confirm the presence of the viruses and viroids identified by NGS (Table S11). In fact, it was very difficult for us to distinguish very closely related viruses such as ACLSV and APCLSV, and APV1 and APV2 with only transcriptome data. Several newly designed primers based on known reference genomes were tested, and of them, selected primer-pairs showing virus specific amplification were used for RT-PCR. The RT-PCR results showed that ACLSV was infected all cultivars except JT (Figs 8a and S5). APCLSV and CNRMV were infected in BC and MB, respectively. APV2 and PcVT were infected in both BC and JH, respectively. PLMVd was infected in all examined peach cultivars, while HSVd was infected in all cultivars except JT. In addition, RT-PCR revealed that HSVd was infected in CJ and BC cultivars.

## Discussion

In this study, we studied the peach RNA virome in a single peach plant and viral communities in six different cultivars using metatranscriptome analyses. Of identified six viruses and two viroids, five viruses were assigned as members in the family *Betaflexiviridae*, which includes two genera, *Foveavirus* (APV1 and APV2) and *Trichovirus* (ACLSV and APCLSV) (Fig. 8b). Moreover, PcVT, a novel virus that is tentatively a member of the family *Tymoviridae*, was identified. Although several studies based on NGS have reported the identification of known or novel viruses in many plant species^11,34,35^, our study differs from previous reports in many aspects. Here, we addressed or discussed several key points derived from this study associated with plant virome and viral communities.

Peach trees are usually propagated by grafting. Therefore, many viruses and viroids may be transmitted through scions and rootstocks. In Korea, peach seedlings are normally used for the rootstocks of peach trees. In this study, all rootstocks were peach seedlings derived from a single wild peach tree grown on a mountain without human contact. Furthermore, we have confirmed that this wild peach tree had not been infected by any viruses and viroids by using NGS and RT-PCR. Therefore, we could be certain that viruses and viroids were not transmitted from rootstocks in our study.

The emergence of several NGS systems is an innovation in many research areas including virology^11,35^. In general, the first step of virus identification is to observe the disease symptoms caused by viruses^12^. However, viral infection in plants does not always cause disease symptoms. The disease symptoms might depend on the virus, host, and environmental conditions. The peach trees used in this study did not always show virus disease symptoms, which indicates the importance of environmental conditions such as cultivation method, climate, pesticides, and fertilizers. In addition, disease symptoms were highly dependent on the host. For example, only BC and JH out of the six cultivars displayed disease symptoms.

A recent study has suggested possible effects of the virome with regard to host health and disease^36^. However, the complex of the virome in a single tree made it difficult to determine the major virus or viroid that might be associated with disease symptoms. Several studies have shown that the coinfection of several viruses could create competition of mutually beneficial cooperation^37,38^. Furthermore, the coinfection of PNRSV and PLMVd resulted in synergistic effects at the transcriptomic level of peach fruits^39^. We supposed that at least closely related viruses can be beneficial for replication, movement, and pathogenesis due to their common characteristics. However, the impact of coinfection among entirely different viruses and viroids in the viral lifecycle and the host should be studied. Moreover, we found that the coinfection of different viruses in the host did not always correlate with disease symptoms, as shown in the high quality fruits obtained from the six cultivars.

The preparation of a library for NGS is a very important factor for virome study. Many recent studies have preferentially prepared libraries from ribosome-deleted^13^, small RNAs^40^, and extracted double-stranded (ds) RNAs^34^. Theoretically, NGS using a ribosome-deleted library possibly identifies both poly-A tailed viruses and dsRNA without poly-A tail and viroids with circular RNA genomes. However, our recent studies have demonstrated that the mRNA library prepared by oligo dT is also adaptable enough to identify different types of viral genomes such as DNA viruses, double stranded RNA viruses, and viroids regardless of the presence of poly-A in the genome^24–26^. A recent study found that a wide range of RNA viruses contain nontemplated oligo(U) or (U)-rich tails, suggesting widespread 3′ uridylation in eukaryotic viruses^41^. Thus, we carefully supposed that some (U)-rich regions in viruses without poly-A tail can be amplified by oligo dT, which is used for mRNA preparation. Of course, the mRNA based library might be not useful to amplify viruses without poly-A tail; however, the mRNA libraries in this study were successfully applied in *de novo* assembly of 12 virus genomes with poly-A tail. In addition, we confirmed the absence of *Little cherry virus 1* and *Peach mosaic virus* in the family *Closteroviridae* which do not possess poly-A tail in the six peach cultivars by RT-PCR. Therefore, we carefully suggested that the library type should be dependent on the purpose of the study.

The pooling of samples might increase the number of identified viruses, but this would make obtaining the detailed information of the virome in a single plant impossible. Prior to conducting RNA-Seq, we checked the infection of known viruses and viroids by RT-PCR. This approach enabled us to compare different viromes from individual single peach cultivars. Sometimes, NGS did not detect viruses or viroids in low titer, even though RT-PCR could detect them. In addition, in the preparation of the six libraries, we added barcode sequences to each library and the pooled libraries were sequenced using a HiSeq. 2000 system. Technically, the individual barcoded sequences should be sorted during data analysis, but we found that some identified viruses with small number of reads should be carefully analyzed. Sometimes, they might contaminate sequences derived from other libraries. Thus, a combination of at least two different approaches was necessary for virus identification and diagnosis.

A complex mixture of viruses in a single plant was an obstacle to distinguishing closely related viruses. For example, NGS results showed the coinfection of APV1 and APV2 in JH, and the coinfection of ACLSV and APCLSV in BC. Complete or nearly complete genome sequences are very useful, as described previously^30,42^, for determining closely related genomes. The assembled contigs associated with identified viruses in our study were sufficient to cover most regions of the target virus. In addition, the information of available reference genome sequences was beneficial for virus genome assembly.

Compared to previous studies, our study precisely revealed the virome in a single tree and elucidated the abundance of individual viruses or viroids in a single tree by viral reads, copy number, and FPKM values. In fact, it was difficult to determine the abundance of viral RNAs due to the natural mutation of RNA viruses and viroids. We used assembled viral genome sequences generated from the corresponding transcriptome for the calculation of viral abundance and SNPs to minimize possible mismatches caused by mutations. Furthermore, we carefully suggested that FPKM values might be a good tool for the calculation of viral abundance using transcriptome data.

Viral quasispecies, which are a collection of diverse variants, contribute to the characteristics of the viral population^43^. In particular, the advance of NGS techniques facilitates the identification of virus SNPs using sequenced data. The analyses of SNPs revealed the mutation frequency for each identified virus or viroid. As previously reported^44^, the 240 PLMVd genomes in this study showed strong genetic variations, whereas the HSVd genomes were relatively conserved. Out of the examined RNA viruses, the sequences of five ACLSV isolates showed a low rate of SNP, whereas APV1, APV2, CNRMV, and PeVD showed relatively higher rates of SNPs. Two closely related PeVD and PcVT showed that the SNP frequency of PeVD was very high in BC as compared to that of PcVT with low SNP frequency. This result might indicate a possible host-specific mutation of viral RNA genome.

Taken together, we provide a comprehensive overview of the peach RNA virome in a single tree and different viral communities in different cultivars. We successfully applied intensive bioinformatics analyses in genome assembly, SNPs, abundance of viral RNAs, and phylogenetic analyses. We suppose that several unknown factors including environment and genetic background contributed to the formation of unique features for individual peach viral communities in different cultivars. The elucidation of the possible impacts of individual peach viral communities associated with plant health might be very challenging and complicated due to the complexity of viral communities.

## Methods

### Plant materials

The peach cultivars used in this study were grown in an orchard in Hoengseong, Korea. In total, we used six different peach cultivars, which are widely grown in Korea: Jangtaek (JT), Mibaek (MB), Cheongjung (CJ), Cheonghong (CH), Janghowon (JH), and Baekcheon (BC) for the peach virome study. We used leaf samples from a single plant for individual peach cultivar. In general, the peach cultivars did not show any visible viral disease symptoms. We examined viral disease symptoms in the selected peach plants for the years 2013–2016. JH and BC cultivars showed visible viral disease symptoms in leaves in years 2013 and 2014; however, we could not observe any visible disease symptoms in 2015 or 2016.

### Total RNA extraction

Five leaf samples from a single plant were rapidly frozen in the presence of liquid nitrogen and kept at −70 °C for further experiments. Frozen leaf tissues were ground in liquid nitrogen with a mortar and pestle. We extracted total RNA using two kits: Fruit-mate for RNA Purification (Takara, Shiga, Japan) and the RNeasy Plant Mini Kit (Qiagen, Hilden, Germany) according to manufacturer’s instructions. The quality and quantity of extracted total RNA were checked. Only high quality total RNA was used for the library preparation for RNA-Seq.

### Library preparation for RNA-Seq

Extracted total RNAs were used for the library construction using the TruSeq RNA Library Preparation Kit v2 (Illumina, CA, USA) according to the manufacturer’s instructions. In this study, we generated six mRNA libraries derived from six different plants. In brief, mRNAs with poly-A tail were extracted using poly-T oligo-attached magnetic beads. The purified mRNAs were subjected to the first strand of cDNA followed by a second strand of cDNA, and the adenylation of 3′ ends was carried out. Adapters were ligated, and PCR amplification was performed to selectively enrich DNA fragments with adapters and amplify the amount of DNA in the library, respectively. We conducted quality control for the generated libraries using a 2100 Bioanalyzer (Agilent, Santa Clara, USA). The libraries were paired-end sequenced by Macrogen Co. (Seoul, South Korea) using the HiSeq 2000 platform.

### Assembly of *de novo* transcriptome

We used the raw data obtained from six different libraries for *de novo* transcriptome assembly. We subjected two paired-end sequenced FASTQ files for each individual library to *de novo* transcriptome assembly using the Trinity program (version 2.0.2, released 22^nd^ January 2015) with default parameters^27^. We used a workstation with two six-core CPUs and 256 GB RAM operated using the Ubuntu 12.04.5 LTS operation system for most bioinformatics analyses including *de novo* transcriptome assembly and BLAST search. In addition, we used the Velvet/Oases assembler (version 0.2.08)^28^ for the *de novo* transcriptome assembly to complement results from the Trinity program.

### BLAST search to identify viruses and viroids

We used the MEGABLAST algorithm^45^ with a cut-off E-value of 1e^−5^ to identify viruses and viroids in assembled peach transcriptomes. We combined sequences for plant viruses and viroids from the complete reference sequences of viruses and viroids (http://www.ncbi.nlm.nih.gov/genome/viruses/) and descriptions of plant viruses (http://www.dpvweb.net/) to establish a specific database for plant viruses and viroids^46^. We blasted the assembled contigs from each peach transcriptome against a database composed of plant viruses and viroids. The identified contigs associated with viruses and viroids were extracted and used for a tBLASTx search against a NCBI NR (non-redundant proteins) database. After filtering endogenous virus-like sequences, only clean contigs associated with viruses and viroids were obtained.

### The *de novo* genome assembly for identified viruses and viroids

We conducted *de novo* transcriptome assembly for individual peach cultivar by two different programs to assemble complete or nearly complete viral genome for the identified viruses and viroids: Trinity and Velvet. Based on our previous study^24,26^, both programs have advantages and disadvantages for the transcriptome assembly. The best way is to combine the contigs associated with viruses and viroids for viral genome assembly. Viral contigs were aligned on the corresponding virus genome using ClustalW program implemented in the MEGA7 program for *de novo* assembly followed by BLAST search^47^. In many cases, we could fill gaps in a viral genome with contigs obtained from Velvet. Moreover, we again mapped raw sequence reads on the assembled viral genome to confirm consensus sequences using a Burrows-Wheeler Aligner (BWA) program with default parameters^48^. The open reading frames (ORFs) for known viruses were analyzed based on the respective reference viral genome. In the case of PeVD and PcVT, ORFs were predicted by ORFfinder (https://www.ncbi.nlm.nih.gov/orffinder/) and conserved domains were analyzed by SMART program (http://smart.embl-heidelberg.de/)^49^.

### Construction of phylogenetic trees

We generated phylogenetic trees for viruses in which the genome sequences were complete or nearly complete in this study. Sequence similarity between ACLSV and APCLSV genomes meant that we could use five ACLSV genomes (isolates CH, CJ, BC, JH, and MB) and an APCLSV isolate BC for the construction of phylogenetic tree. In a similar manner, a genome for APV1 isolate JH and two genomes for APV2 isolates BC and JH were used for the construction of a phylogenetic tree. In total, 18 ACLSV genomes, two APCLSV genomes, five APV1 genomes, six APV2 genomes, three APV3 genomes, and six CNRMV genomes were used for phylogenetic analyses. Three CTLV genomes were used as outgroup in the CNRMV phylogenetic tree. The accession number for each viral genome was indicated in the phylogenetic tree. We conducted BLAST using the identified virus as a query against GenBank (http://www.ncbi.nlm.nih.gov/genbank/) to reveal phylogenetic relationships with other known viruses. After filtering partial sequences, we only retrieved complete genome sequences homologous to each virus from the nucleotide database. We aligned the genome sequences for each virus using the ClustalW program with default parameters. After sequence alignment, unnecessary sequences and poly-A tails at the 5′ and 3′ regions, respectively, were removed. Aligned sequences were manually edited and used for the construction of a phylogenetic tree using the MEGA7 program^47^. The phylogenetic tree was constructed via the neighbor-joining method with 1,000 bootstrap replicates and Kimura 2-parameter distance.

In the case of HSVd, we amplified complete genomes for five HSVd isolates MB, CJ, CH, JH, and BC via RT-PCR using HSVd-specific primers. The PCR products were cloned into the pGEM-T-Easy Vector (Promega, Wisconsin, USA) and sequenced by the Sanger sequencing method. The accession numbers for HSVd genomes were KX371905–KX371909. We combined five HSVd genomes in this study with 382 HSVd variants from a recent study to construct a phylogenetic tree. In total, we aligned 387 HSVd genome sequences, and converted the aligned sequences file into a nexus file. The high degree of sequence similarity of HSVd genomes meant that we used the Splitstree program^50^ to show phylogenetic networks rather than other available phylogenetic programs. The generated nexus file was imported into Splitstree to show phylogenetic networks for 387 HSVd genomes.

In the case of PLMVd, we conducted RT-PCR using two different primer-pairs. The amplified PCR products were cloned and sequenced as described for HSVd. Each primer-pair amplified 20 clones. Thus, we sequenced 40 clones for each cultivar. We used 240 PLMVd derived from six different cultivars for PLMVd phylogenetic analyses; again, we used the Splitstree program. We identified ten different groups, and calculated the number of PLMVd isolates in each group.

### Analysis of SNPs using transcriptome data

We analyzed SNPs for only viruses and viroids, in which complete or nearly complete genomes were obtained in this study. We used the same method to analyze SNPs for each viral genome as described previously^25^. In brief, we mapped the raw sequence reads from each transcriptome on the identified individual viral genome using the BWA program with default parameters. We used the assembled viral genome from each transcriptome for reference to increase SNP specificity. In general, the application of known viral reference genomes for the analysis of SNPs led to the identification of unexpected SNPs. We converted the SAM files generated by the BWA program into BAM files via SAMtools^51^. The sorted BAM files were used to generate the VCF file format using the mpileup function of SAMtools for SNP calling. Finally, we used BCFtools implemented in SAMtools to call SNPs. The positions of identified SNPs on each viral genome were visualized by the Tablet program^52^.

### Calculation of virus accumulation and copy number

We used two different approaches to quantify viral RNAs associated with identified viruses and viroids. The first was to calculate the sequenced reads associated with respective viruses or viroids via MEGABLAST search. We converted the FASTQ files into FASTA files using the FASTX-Toolkit (http://hannonlab.cshl.edu/fastx_toolkit/) for this, and we subjected the converted FASTA files to MEGABLAST. The second was the estimation of transcript abundance based on alignment-based abundance estimation methods used for RNA-Seq^33^. We aligned the raw sequence reads on the identified viral contigs using the Bowtie 2 program^53^ and subjected this to the FPKM method to calculate the normalized expression value “fragments per kilobase transcript length per million fragments mapped” (FPKM). In the case of BC and JH cultivars, we combined the FPKM values for each identified virus or viroid to estimate the copy number.

### Data Availability

The raw dataset in this study will be available, upon publication, in the Sequence Read Archive (SRA) repository with the following accession numbers SRR2290949, SRR2290951, SRR5074727, SRR5074715, SRR5074728, and SRR5074714. The viral genome sequences obtained from this study were also deposited in GenBank, NCBI with respective accession numbers.

## Electronic supplementary material

Supporting information

Supplementary File S1-11

Supplementary File S10
