# Supplementary material for: Peach RNA viromes in six different peach cultivars
Source: Sci Rep. 2018 Jan 30;8:1844. doi: 10.1038/s41598-018-20256-w (PMC5789896; doi:10.1038/s41598-018-20256-w)
Supplement: Supplementary file 1 — Supporting information [file 41598_2018_20256_MOESM1_ESM.pdf]

Supplementary Information

Peach RNA viromes in six different peach cultivars

Yeonhwa Jo[1], Sen Lian[2], Hyosub Chu[1], Jin Kyong Cho[3], Su-Hyun Yoo[1], Hoseong Choi[1], Ju-Yeon Yoon[4], Seung-Kook Choi[4], Bong Choon Lee[5], and Won Kyong Cho[1,3]*

[1]Department of Agricultural Biotechnology, College of Agriculture and Life Sciences, Seoul National University, Seoul, 08826, Republic of Korea

[2]College of Crop Protection and Agronomy, Qingdao Agricultural University, Qingdao, Shandong 266109, China

[3]The Taejin Genome Institute, Hoeongseong, Gadam-gil 61, 25239, Republic of Korea

[4]Virology Unit, Department of Horticultural Environment, National Institute of Horticultural and Herbal Science, RDA, Wan-Ju, 565-852, Republic of Korea

[5]Crop Foundation Division, National Institute of Crop Science, RDA, Wanju, 55365, South Korea

Supplementary data

Supplementary Tables

Table S1. Summary of raw data for the individual library sequenced by the HiSeq 2000 system.

Table S2. Summary of de novo transcriptome assembly for six peach transcriptomes.

Table S3. Virus-associated contigs identified by MEGABLAST against the viral genome database.

Table S4. The list of identified viruses and viroids from six peach cultivars.

Table S5. The de novo assembled viral genomes using peach transcriptome data.

Table S6. BLAST results using assembled genomes for Peach virus T isolate BC and JH against the nucleotide database (NT) in NCBI.

Table S7. BLAST results using assembled genomes for ACLSV and APCLSV against nucleotide database (NT) in NCBI.

Table S8. BLAST results using assembled genomes for APV1 and APV2 against nucleotide database (NT) in NCBI.

Table S9. BLAST results using an assembled genome for CNRMV isolate MB against nucleotide database (NT) in NCBI.

Table S10. Analysis of single nucleotide variations for individual viral genomes in a single plant.

Table S11. Detailed information for the primers used in this study.

Supplementary Figures

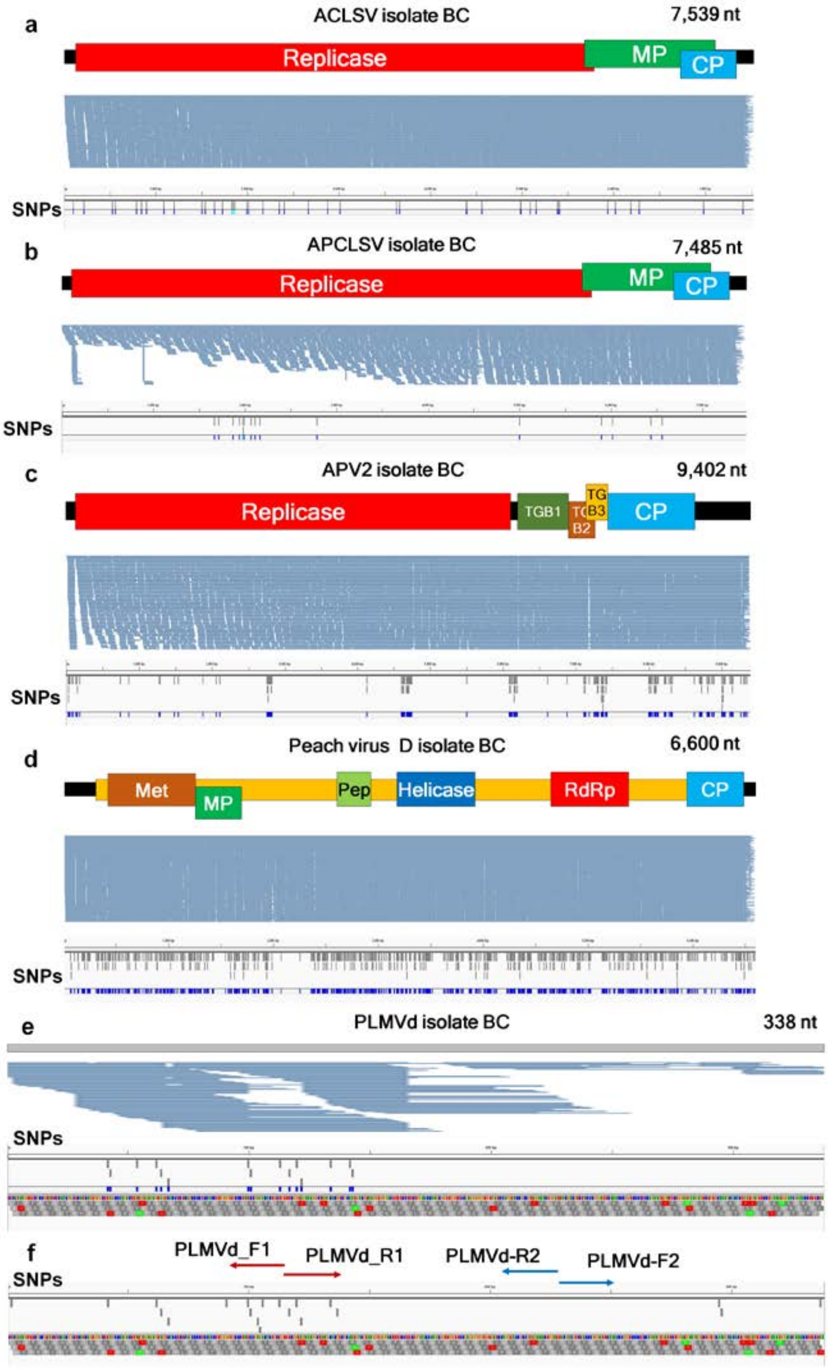

Figure S1. Single nucleotide variations of identified viruses and viroids in the BC cultivar. The genome organization, alignment of sequenced reads, and identified SNVs for ACLSV isolate BC (a), APCLSV isolate BC (b), APV2 isolate BC (c), and PcVT isolate BC (d). The blue bars indicate the position of identified SNVs on the given virus genome. (e) The alignment of sequenced reads and identified SNVs for PLMVd isolate BC. (f) The identified SNVs in the BC cultivar using 40 PLMVd genome sequences, which were amplified by two different primer pairs followed by Sanger Sequencing. Red and blue arrows indicate the positions of two primer-pairs for RT-PCR.

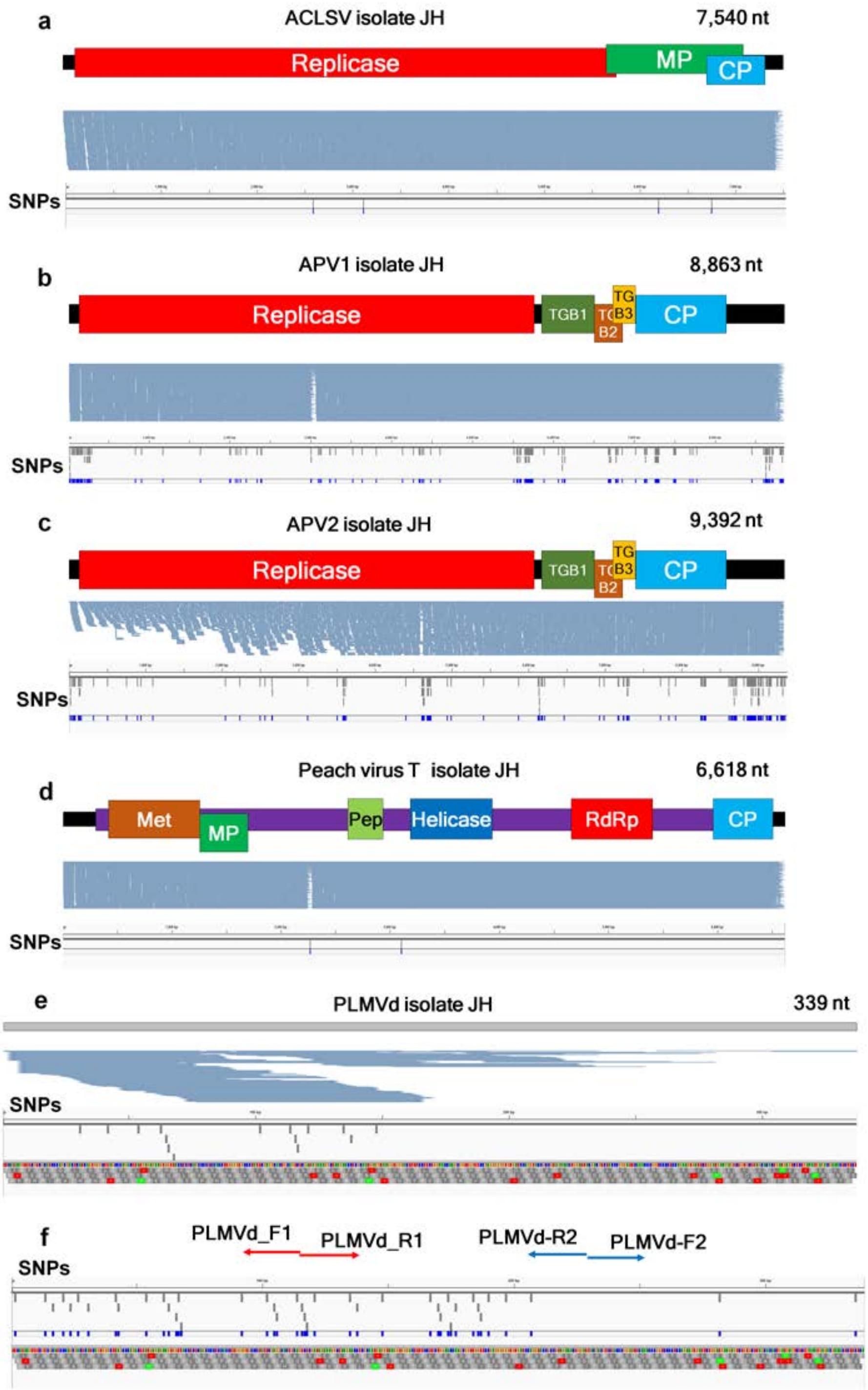

Figure S2. Single nucleotide variations of the identified viruses and viroids in the JH cultivar. Genome organization, alignment of sequenced reads, and identified SNVs for ACLSV isolate JH (a), APCLSV isolate JH (b), APV2 isolate JH (c), and PcVT isolate JH (d). Blue bars indicate the position of identified SNVs for the given virus genome. (e) The alignment of sequenced reads and identified SNVs for PLMVd isolate JH. (f) The identified SNVs in the JH cultivar using 40 PLMVd genome sequences, which were amplified by two different primer pairs followed by Sanger Sequencing. Red and blue arrows indicate the positions of the two primer pairs for RT-PCR.

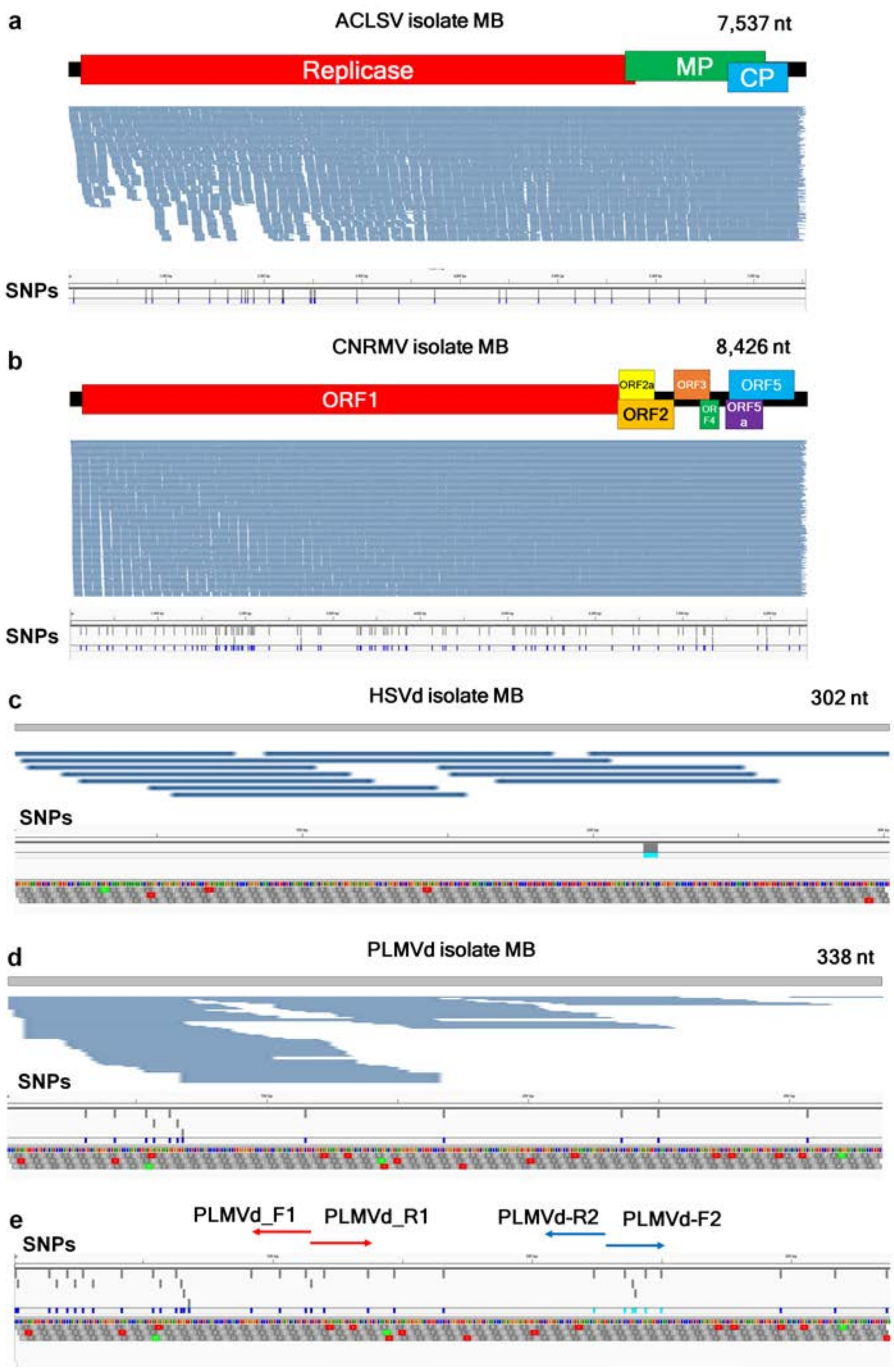

Figure S3. Single nucleotide variations of identified viruses and viroids in the MB cultivar. Genome organization, alignment of sequenced reads, and identified SNVs for ACLSV isolate MB (a), CNRMV isolate MB (b). Blue bars indicate the position of identified SNVs on the given virus genome. (c) Alignment of sequenced reads and identified SNVs for HSVd isolate MB and PLMVd isolate MB (d). In the case of HSVd and PLMVd, sequenced reads were aligned on a single circular RNA genome. (e) Identified SNVs in the MB cultivar using 40 PLMVd genome sequences that were amplified by two different primer pairs followed by Sanger Sequencing. Red and blue colored arrows indicate the positions for two primer-pairs for RT-PCR.

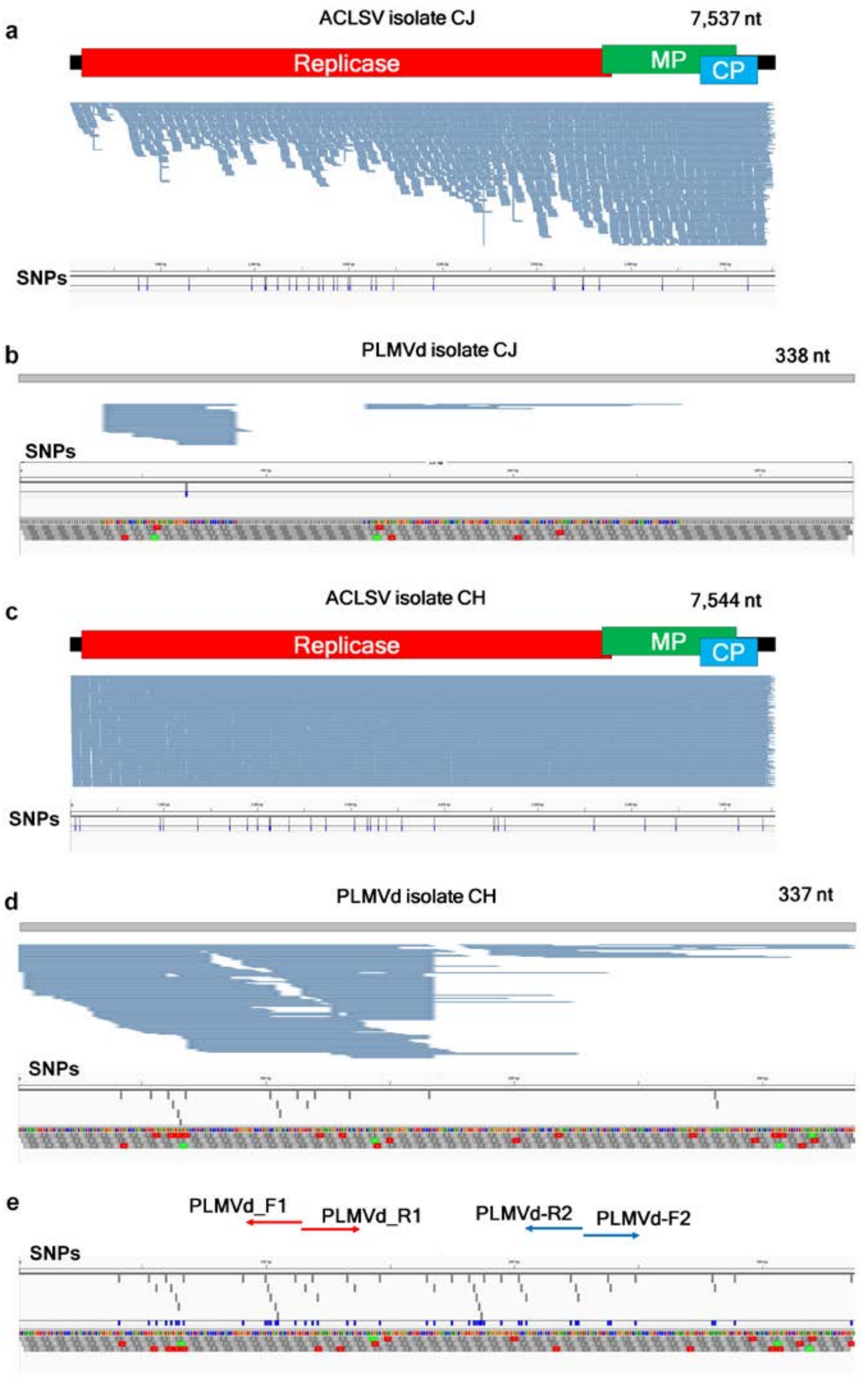

Figure S4. Single nucleotide variations of identified viruses and viroids in the CJ and CH cultivars.
(a) Genome organization, alignment of sequenced reads, and identified SNVs for ACLSV isolate CJ. (b) Alignment of the sequenced reads and identified SNVs for PLMVd isolate CJ. (c) Genome organization, alignment of sequenced reads, and identified SNVs for the ACLSV isolate CH. (d) Alignment of sequenced reads and identified SNVs for the PLMVd isolate CH. (e) Identified SNVs in the CH cultivar using 40 PLMVd genome sequences that were amplified for two different primer pairs followed by Sanger Sequencing. Red and blue colored arrows indicate the positions of two primer-pairs for RT-PCR.

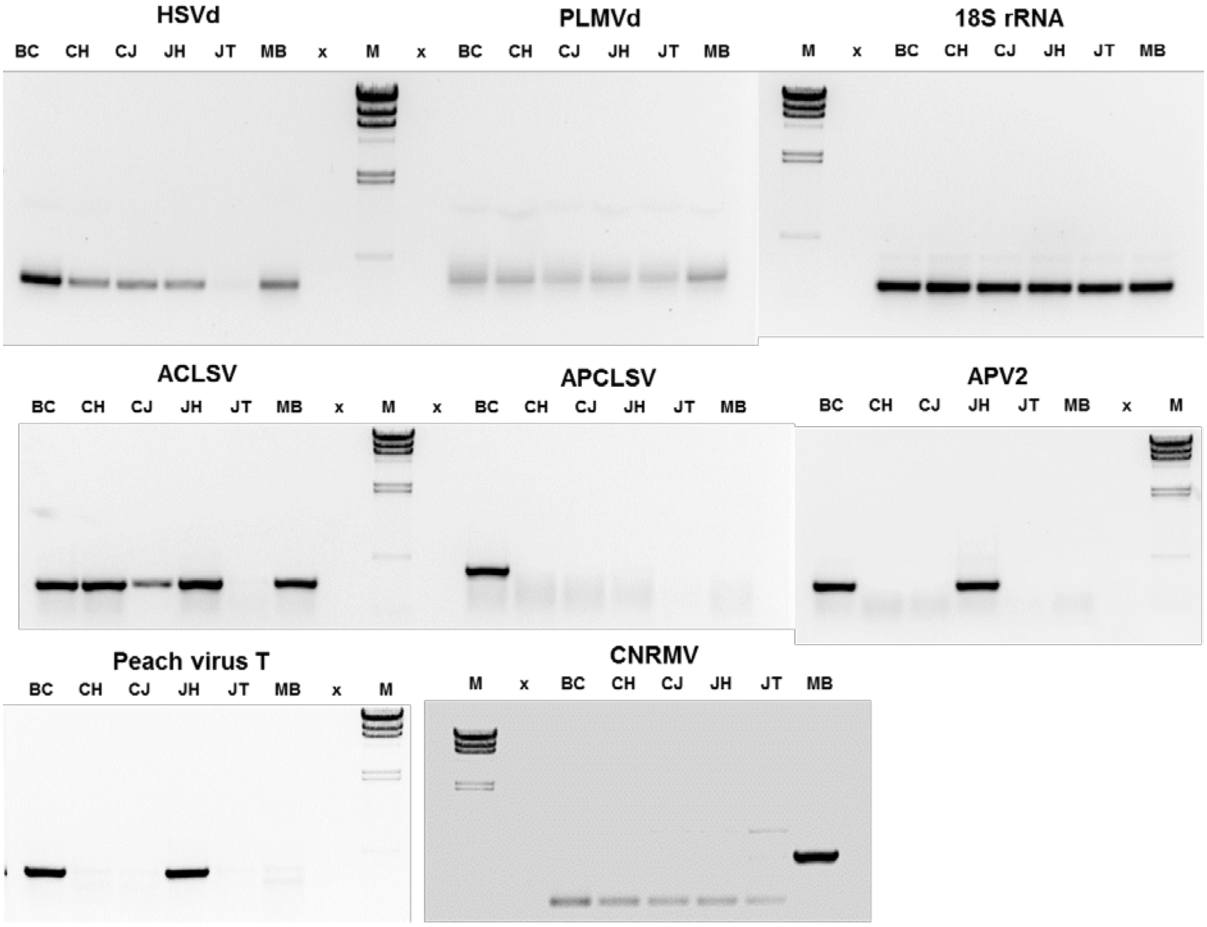

Figure S5. Full-length gels display RT-PCR results with virus- and viroid-specific primers. The 18S rRNA gene was used as a positive control.
